# Age-related and disease-specific changes in B-cell profiles in older adults with immune thrombocytopenia

**DOI:** 10.3389/fimmu.2026.1791460

**Published:** 2026-05-12

**Authors:** E. Monzón Manzano, C. Herrero Carrasco, P. Acuña, L. del Pino Molina, E. G. Arias-Salgado, A. Mendoza, R. Kapur, L. Porcelijn, M. Martín Salces, M. I. Rivas Pollmar, E. López-Granados, V. Jiménez Yuste, M. Gasior Kabat, R. Ramírez Martín, N. Butta, M. T. Álvarez Román

**Affiliations:** 1 Haematology and Haemotherapy Department, La Paz University Hospital, Bleeding and Haemostasis Disorders Group-Health Research Institute of the La Paz University Hospital (IdiPAZ), Madrid, Spain; 2 Haematology and Haemotherapy Department, Severo Ochoa University Hospital, Madrid, Spain; 3 Clinical Immunology Department, La Paz University Hospital, and Center for Biomedical Network Research on Rare Diseases (CIBERER U767), Madrid, Spain; 4 Sanquin Blood Supply Foundation, Research Department, and Amsterdam University Medical Center (UMC) location, Landsteiner Laboratory, Amsterdam, Netherlands; 5 Sanquin Diagnostic Services, Department of Immunohematology Diagnostics, Sanquin, Amsterdam, Netherlands; 6 Lymphocyte Pathophysiology in Immunodeficiencies Group-Health Research Institute of the La Paz University Hospital (IdiPAZ), Madrid, Spain; 7 Faculty of Medicine, Universidad Autónoma de Madrid, Madrid, Spain; 8 Geriatrics Department, La Paz University Hospital –Ageing and Fragility in Elderly Persons Group-Health Research Institute of the La Paz University Hospital (IdiPAZ), Madrid, Spain

**Keywords:** aged patients, BAFF, B-cells, immune thrombocytopaenia, naïve B-cells

## Abstract

**Introduction:**

Immune thrombocytopenia is an autoimmune bleeding disorder that is more prevalent among older adults. Ageing itself reduces B-cell counts, a change also observed in patients with ITP. This study examined the B cell profiles of patients with ITP aged over 65 (ITP>65) and aged 65 or under (ITP ≤ 65). These ITP groups were compared with age-matched healthy controls to determine whether the observed differences were due to the disease itself or the effects of ageing.

**Methods:**

Blood samples were processed and stained using the EuroFlow 8-colour PIDOT and pre-germinal centre B-cell tubes, following the EuroFlow SOPs for staining cell surface membrane markers.

**Results:**

Patients with ITP>65, compared with those ≤65, showed reduced immature/transitional B cell subsets, an increased population of CD21-CD24- naïve B cells, and higher plasma B-cell activating factor levels. Comparison of the ITP ≤ 65 group with the HC ≤ 65 group showed that patients with ITP had a lower B-cell count, but a significant increase in the CD21-CD24- naïve B-cell subset. Patients with ITP>65 had expanded CD21-CD24- and CD21-CD24++ naïve and memory IgMD+ B-cell populations compared to *the* HC>65 group.

**Conclusion:**

These findings suggest that ageing induces modifications to the B-cell phenotype that are similar to those observed in patients with ITP, except for the expansion of the CD21-CD24- naïve B-cell subset, *which appears to be a characteristic of ITP shared with other autoimmune diseases.*

## 1 Introduction

Immune thrombocytopenia (ITP) is an autoimmune disorder in which immune-mediated mechanisms lead to reduced circulating platelet counts and an increased bleeding tendency (1).

While the initial event(s) leading to anti-platelet autoimmunity remain unclear, there is strong evidence that autoantibodies and autoreactive CD8+ cytotoxic T cells trigger enhanced platelet destruction and impair the production of platelets by megakaryocytes in the bone marrow. This has been described as a deterioration of the regulatory compartment (Tregs and Bregs) of the immune system in these patients, alongside a polarisation of the response towards Th1 and Th17 cells. Abnormal T lymphocyte function leads to the proliferation and differentiation of self-reactive B lymphocytes. Antiplatelet antibodies facilitate the phagocytosis of platelets by macrophages in the spleen. However, the absence of antibodies in many patients suggests that other mechanisms are involved in the disappearance of platelets from circulation (2).

The complement-mediated destruction of platelets or the desialylation of platelets, followed by their clearance in the liver, have also been proposed as additional mechanisms involved in the pathogenesis of ITP. Given the pivotal role of B and T cells in ITP pathogenesis, a number of immunosuppressive drugs targeting these mechanisms are currently in use or being studied in clinical trials (3–6).

Numerous epidemiological studies have shown that the incidence of ITP increases after the age of 60, reaching its peak in patients over 80 (7). This may be explained by the fact that all organ systems undergo physiological changes with ageing, including the immune system. The ageing process is associated with a less effective immune system over time, a phenomenon known as immunosenescence (8–10). This is due to the immune system continuously adapting to an ever-expanding range of environmental exposures throughout life. This involves multiple changes, such as modifications to the structure and antigenicity of self-antigens, as well as quantitative and phenotypic abnormalities of lymphocytes and antibodies. These include a reduction in precursor and naïve B-cells, and impaired antibody responses (11). The mechanism by which B-cell tolerance is lost remains unclear. However, elevated levels of B-cell activating factor (BAFF) are likely to be an important contributing factor as they drive B-cell maturation (12). BAFF promotes B-cell survival and increases immunoglobulin production by binding to surface B-cell receptors (13).

According to the World Health Organization, ageing is typically defined by chronological age. Conventionally, a person over the age of 65 is often referred to as elderly (14).

This study aimed to expand existing knowledge of B-cell-related immune dysregulation in ITP and investigate whether ageing has an additional effect on this. To this end, B-cell compartments were examined using flow cytometry, and B-cell distribution was analysed in ITP patients aged over 65 (ITP>65) compared with ITP patients aged 65 or under (ITP ≤ 65). We also compared the ITP groups with age-matched healthy controls (HC) to determine whether any observed differences are attributable to the disease itself or to the effects of ageing.

## 2 Methods

### 2.1 Study design

We conducted an observational and prospective study including adult patients with chronic primary ITP, who were stratified into two age groups: ITP ≤ 65 [n=40; 67.5% female, mean age 43 ± 15, mean platelet count (range) 147 (10-685) x10^3^ platelets/µL] and ITP>65 [n=40, 62.5% female, mean age 77 ± 7, mean platelet count (range) 197 (20-1591) x10^3^ platelets/µL]. The standard reference ranges for the HC ≤ 65 group were obtained from 60 individuals [51% female, mean age 54 ± 15, mean platelet count 245 (range) (105-431) x10^3^ platelets/µL], described in del Pino Molina et al., 2020 (15); and for the HC>65 group, from 40 individuals [57% female, mean age 78 ± 9, mean platelet count (range) 244 (124-663) x10^3^ platelets/µL].

Patients were excluded if they had received B-cell depletion therapy within three years of the study, or who were under treatment with coagulation- and/or platelet-active drugs, or had undergone blood transfusion within 15 days of the study.

Ethics approval was obtained from the La Paz University Hospital Ethics Committee (PI-5524) and signed informed consent was obtained from the participants prior to conducting this study.

During the preparation of this work the author(s) used free versions of DeepL Write in order to improve the quality of the English writing. After using this tool, the author(s) reviewed and edited the content as needed and take(s) full responsibility for the content of the publication.

### 2.2 Phenotypic B-cell analysis

Blood samples collected in EDTA were processed and stained using the EuroFlow 8-colour PIDOT and pre-germinal centre (GC) B-cell tubes, following the EuroFlow SOPs for staining cell surface membrane markers. Details of the specific antibody clones and fluorochrome-conjugated reagents used are provided in **Supplementary Table 1**.

For data analysis, a standardised gating strategy was used for the identification of all pre-GC (defined as CD19+ CD27*−* sIgM+ B-lymphocytes) and post-GC B-cell subsets (defined as CD19+ CD27+ or CD19+ CD27*−* smIgM- B-cells). The pre-GC B cells comprise three subsets of maturation-associated immature B-cells and three subpopulations of mature naïve B lymphocytes. The immature/transitional B-cells include CD5*−*CD38++CD21hetCD24++, CD5+CD38+/++CD21hetCD24++ and CD5+CD38hetCD21+CD24+ immature B cells). The mature naïve B-cells comprise CD21+CD24+, CD21*−*CD24*−*, and CD21*−*CD24++. Post-GC B cells include unswitched and switched IgMD- memory B-cells and plasmablasts/plasma cells. Samples were analysed on a FACSCanto II flow cytometer (Becton/Dickinson Biosciences (BD), Madrid, Spain) using Infinicyt software (BD, Madrid, Spain). For each B-cell population, absolute counts were calculated from the white blood cell (WBC) count in a conventional haematological cell counter, and the percentage of total B-cells obtained with the PIDOT tube for the same sample, as previously reported (15, 16).

### 2.3 Measurement of plasma level of BAFF and of antiplatelet antibodies

BAFF level was measured in plasma using the ELISA kit from R&D Systems Europe Ltd (Spain). Anti-platelet antibodies (anti-GPIIb/IIIa, anti-GPV and anti-GPIb/IX) were measured in serum samples using MAIPA (monoclonal antibody immobilisation of platelet antigens) assays (17).

### 2.4 Statistical analyses

The sample size required to detect differences between the ITP ≤ 65 and ITP>65 groups was calculated using G*Power (version 3.1.9.7) with an alpha level of 0.05 and a power of 0.80. The size effect was calculated based on the means and standard deviations obtained in preliminary experiments. Thus, 22 individuals per group were needed for the analysis of naïve CD21*−*CD24*−* B-cells.

Statistical analyses were performed using GraphPad Prism software 6.0. Depending on data distribution, results were expressed as median (percentile 25%–percentile 75%) or mean ± standard deviation (SD) and comparisons were carried out using parametric (Student’s t-test) or non-parametric (Mann-Whitney) tests. All tests were two-tailed and statistical significance was set at p<0.05.

*The p-values were interpreted descriptively without adjusting for multiple comparisons. Therefore, given the number of B-cell subsets analysed, there is a greater chance of committing a Type I error. Consequently, our findings should be viewed as hypothesis-generating rather than confirmatory. As this was an exploratory study, confirmation of the results in independent cohorts with the appropriate correction for multiple comparisons is required.*

## 3 Results

### 3.1 Demographics of patients with ITP and clinical data

A higher proportion of patients in the ITP ≤ 65 group were not receiving treatment for ITP at the time of sampling compared with those in the ITP>65 group. The most commonly used drugs in both groups of ITP patients receiving treatment were thrombopoietin receptor agonists (TPO-RAs). Among the ITP ≤ 65 group, 57.5% were receiving only TPO-RAs; whereas in the ITP>65 group, 67.5% were receiving TPO-RAs alone and 12.5% were receiving them in combination with other drugs (**Table 1**).

**Table 1:** Treatment received by patients with ITP at the time of sample collection, the number of previous lines of treatment received for ITP and the number of splenectomised patients in each ITP patient cohort.

Treatment at sampling		+IVIg	+ ROMI	+ AVA	+ CORT	CORT + AVA +IVIg	Previous treatments for ITP	
ITP ≤ 65								
WITHOUT, n (%)	9 (22.5)						1, n (%)	7 (17.5)
CORT, n (%)	3 (7.5)	1 (2.5)					2, n (%)	9 (22.5)
ETP, n (%)	11 (27.5)						3, n (%)	12 (30.0)
ROMI, n (%)	8 (20.0)						4, n (%)	9 (22.5)
AVA, n (%)	4 (10.0)						>4, n (%)	3 (7.5)
FOST, n (%)	1 (2.5)		3 (7.5)				Spl, n (%)	6 (15.0)
ITP>65								
WITHOUT n (%)	4 (10.0)						1, n (%)	6 (15.0)
CORT, n (%)	2 (5.0)			1 (2.5)			2, n (%)	14 (35.0)
ETP, n (%)	16 (40.0)						3, n (%)	11 (27.5)
ROMI, n (%)	9 (22.5)						4, n (%)	5 (12.5)
AVA, n (%)	2 (5.0)						>4, n (%)	4 (10.0)
FOST, n (%)	1 (2.5)		3 (7.5)		1 (2.5)	1 (2.5)	Spl, n (%)	2 (5.0)

IVIg, intravenous immunoglobulins; CORT, corticosteroids; ETP, eltrombopag; ROMI, romiplostim; AVA, avatrombopag; FOST, fostamatinib; Sp, splenectomized.

The number of previous lines of treatment was similar in both groups of ITP patients. However, more patients in the ITP ≤ 65 group underwent splenectomy (**Table 1**).

### 3.2 Distribution of B-cell subsets in ITP ≤ 65 and ITP>65 patients

**Figure 1:**
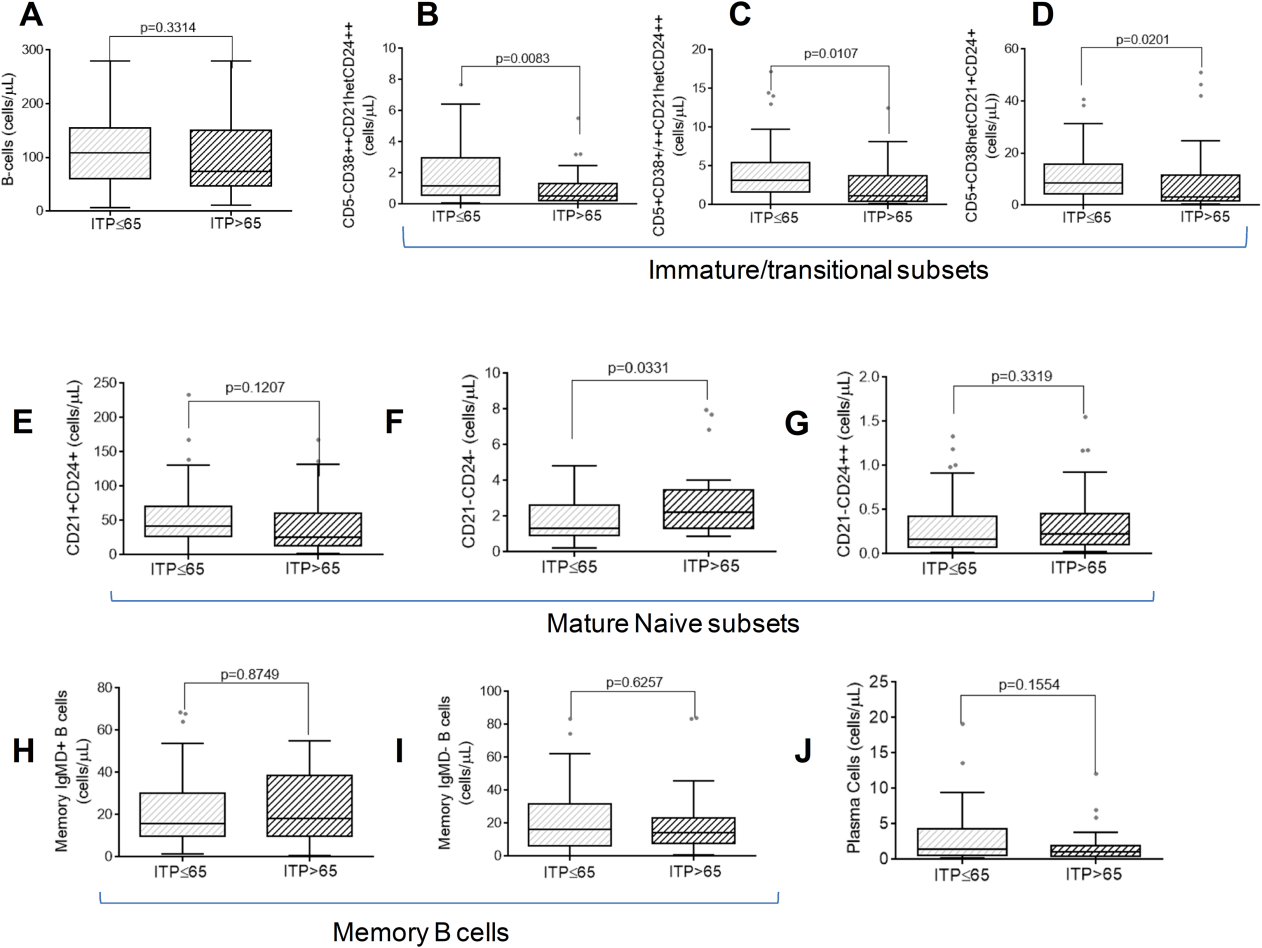
Distribution of distinct subsets of pre- GC, germinal centre and post-GC B-cells was determined by flow cytometry. A comparison was made between ITP ≤ 65 yo and ITP>65 yo in: **(A)** absolute B-cell counts; **(B-D)** immature/transitional B-cell counts; **(E–G)** naïve B-cell counts; **(H, I)** memory B-cell counts; and **(J)** plasma-cell counts. A Mann–Whitney test was performed on the ITP ≤ 65 yo and ITP>65 yo groups, and a p-value<0.05 was considered significant.

Patients with ITP aged ≤65 and >65 years had similar B-cell counts (**Figure 1A**). However, patients over the age of 65 exhibited a decline in immature/transitional B cells when compared to those aged ≤65, which was attributable to a reduction in the number of the following immature B cell subsets: CD5*−*CD38++CD21hetCD24++, CD5+CD38+/++CD21hetCD24++ and CD5+CD38hetCD21+CD24+ (**Figures 1B–D**). Conversely, the naïve B CD21*−*CD24*−* subset was elevated in patients with ITP over 65 years of age compared with those under 65 years of age (**Figure 1F**). No differences were observed between the two ITP groups in the CD21+CD24+ and CD21*−*CD24++ naïve B-cell subsets (**Figures 1E, G**), the non-switched IgMD+ (**Figure 1H**), the switched IgMD- (**Figure 1I**), and plasma B cells (**Figure 1J**).

**Figure 2:**
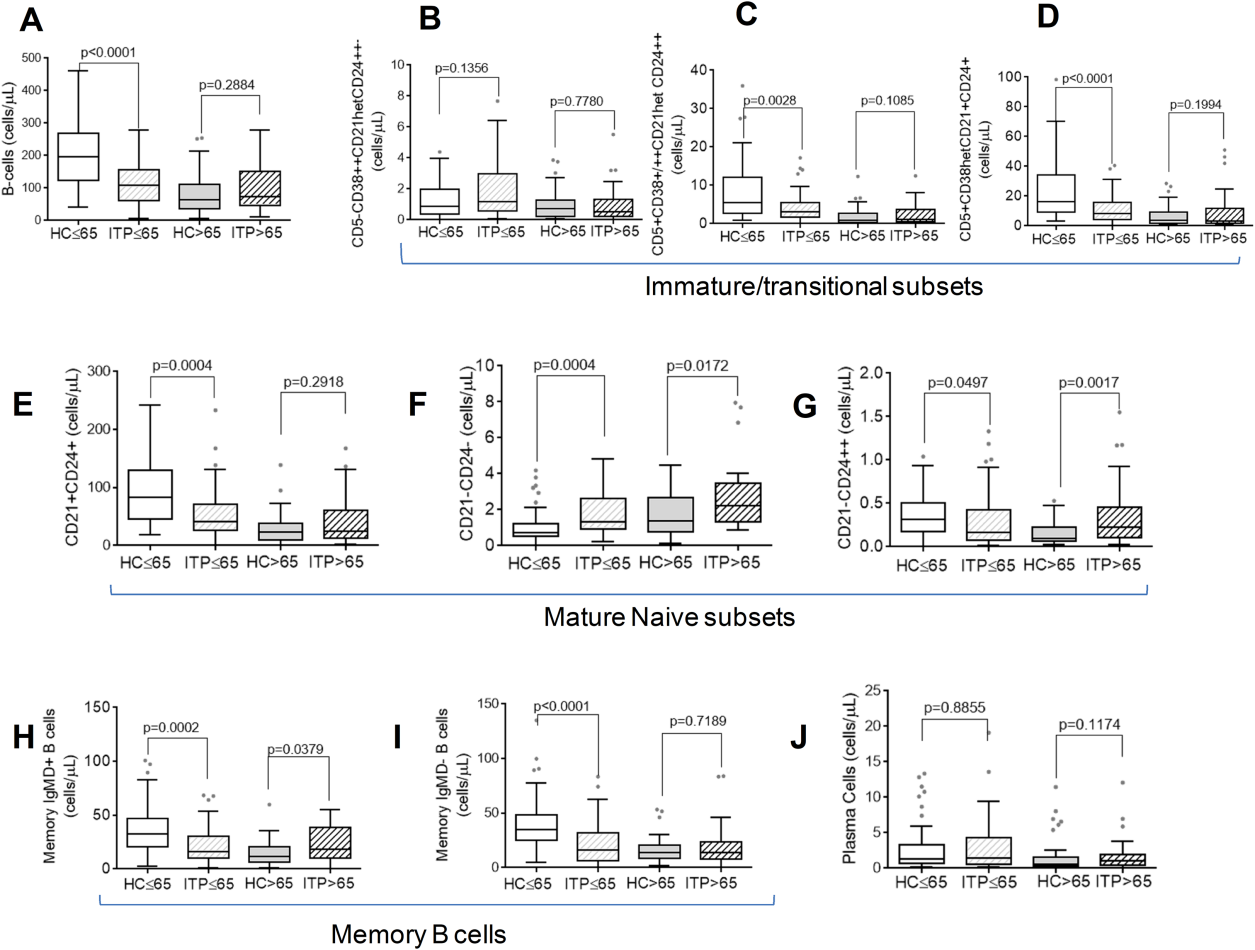
The distribution of distinct subsets of pre- GC, germinal centre and post-GC B-cells was determined by flow cytometry. **(A)** comparison was made between ITP patients aged ≤65 and >65 years and their corresponding age-matched HC, healthy controls. HC Absolute counts of B-cells; **(B–D)** Immature/transitional B-cells; **(E–G)** Mature naïve B-cells; **(H, I)** Memory B-cells; and **(J)** Plasma cells. The Mann–Whitney test was performed for each ITP group compared to their corresponding age-matched HC, and a p-value of less than 0.05 was considered significant.

Patients with ITP aged ≤65 years had a significant reduction in total B cell count compared to age-matched HC. No differences were found between the HC>65 and ITP>65 groups (**Figure 2A**). This decline in B-cells in patients with ITP aged ≤65 years may be due to a reduction in the following immature B-cell subsets: CD5+CD38+/++CD21hetCD24++, and CD5+CD38hetCD21+CD24+ (see **Figures 2C, D**, respectively); the CD21+CD24+ and CD21*−*CD24++ naïve B cell subsets (see **Figures 2E, G**, respectively); and the non-witched IgMD+ (see **Figure 2H**) and switched IgMD- (see **Figure 2I**) subsets.

An increase in the CD21*−*CD24*−* naïve B-cell population was observed in both ITP patient groups compared to their respective HC groups (**Figure 2F**). The ITP>65 group also showed a significant increase in CD21*−*CD24++ naïve B-cells (**Figure 2G**) and memory IgMD+ cells (**Figure 2H**). No other differences were observed between the ITP>65 group and the HC>65 group in the other B-cell subsets (**Figure 2I** and 2J).

As TPO-RAs are the most widely used therapy in both ITP groups and have the capacity to influence immune homeostasis (18, 19), it is not possible to exclude the potential contribution of treatment-related effects to the observed differences in B-cell subsets. To rule out this possibility, we analysed the distribution of B cells in patients receiving TPO-RA monotherapy in both ITP patient groups. The significant differences found in this new data analysis (see **Supplementary Table 2**) were similar to those seen when all patients in both cohorts were included.

### 3.3 Plasma BAFF levels and antiplatelet antibodies

**Figure 3:**
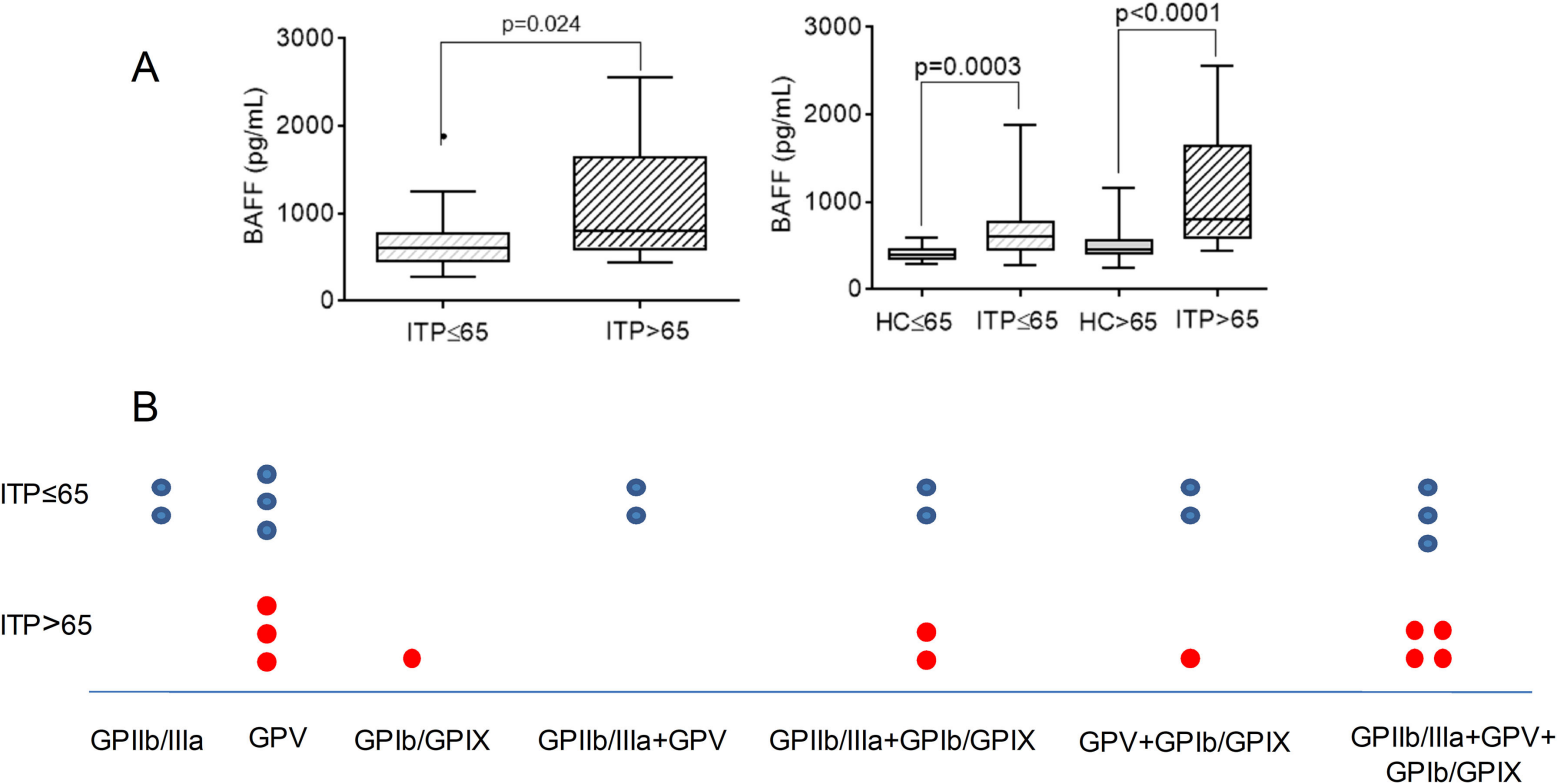
**(A)** Comparison of plasma levels of BAFF between ITP ≤ 65 and ITP>65; and between ITP groups with their age-matched HC, healthy control groups. The Mann–Whitney test was performed for each ITP group *compared to* their corresponding age-matched HC, and a p-value of less than 0.05 was considered significant. **(B)** Each point represents the distribution and specificity of antiplatelet antibodies in patients from ITP ≤ 65 and ITP>65 groups. Each point represents one patient.

Plasma BAFF levels were higher in ITP>65 than in ITP ≤ 65; and higher in both groups of patients with ITP compared to their age-matched HC (**Figure 3A**).

Of the patients with ITP ≤ 65 years old, 35.0% were found to have anti-platelet antibodies in their plasma. Conversely, 27.5% of patients in the ITP>65 years old group exhibited anti-platelet antibody positivity. **Figure 3B** shows the specificity and distribution of antiplatelet antibodies in each age group. No differences were observed in B-cell subsets between the groups without and with antiplatelet antibodies (data not shown).

## 4 Discussion

This study identified differences in the distribution of pre-GC B-cells between patients with ITP>65 and patients with ITP ≤ 65. Patients with ITP>65 had lower numbers of immature/transitional B-cells and higher numbers of CD21-CD24- naïve B-cells than those in the ITP ≤ 65 group. They also had higher plasma BAFF levels.

A higher proportion of patients in the ITP>65 group received pharmacological treatment than those in the ITP ≤ 65 group (90.0% vs 77.5%), which is consistent with previous findings (20, 21). However, overall treatment rates were higher in our cohorts. TPO-RAs were the preferred second-line therapy for all patients with ITP, despite the potential thrombotic risks associated with them, with older patients more frequently treated with TPO-RAs. As TPO-RAs have immunomodulatory effects (19, 22, 23) it is possible that treatment-related factors were responsible for the observed differences in B-cell distribution. However, the fact that the analyses of B-cell distribution between the ITP ≤65 and ITP >65 groups, which only included patients receiving TPO-RA in monotherapy, yielded similar results to those obtained when all ITP patients were included, helps to rule out this possibility. Nevertheless, the observed alterations in B-cell subsets should be interpreted with caution, since B-cell profiles may be affected by previous treatment regimens. It can be challenging to differentiate between the effects of the disease and the effects of the treatment.

The maturation stage of B-cell subpopulations that travel through the blood to different tissues can be classified as immature/transitional, naïve, memory B lymphocytes and plasmablasts/plasma cells. Previous studies have shown different age-related production patterns for B-cell subsets (24).

ITP patients exhibit altered pre-GC B-cells maturation profiles, in particular a decrease in pre-GC B-cells in the earliest stages of maturation (immature/transitional subsets). Research indicates that a higher CD21-CD24- naïve B-cell count is a feature of ITP patients, irrespective of age. This subset of mature B-cells with loss of CD21 expression results from homeostatic stimulation, where they remain in circulation without undergoing GC response and differentiation into memory B-cells, but their functional significance is unclear (25, 26). This is in accordance with previous reports of decreased proportions of total B-cells, and increased naïve B-cells in peripheral blood of patients with ITP (27).

As seen in ITP, the B lymphocyte (LB) compartment with low CD21 expression expands in several autoimmune diseases with chronic immune activation, such as systemic lupus erythematosus (28); rheumatoid arthritis (29); primary Sjögren syndrome (25); systemic sclerosis (30, 31); and anti-neutrophil cytoplasm antibodies–associated vasculitis (32), implying a direct role for these cells in autoimmune pathogenesis. Moreover, common variable immunodeficiency (CVID), the most prevalent symptomatic primary immunodeficiency, is accompanied in 4-19% of CVID patients, by ITP (33). In these patients, the immunophenotypic assessment of B-cells in peripheral blood by flow cytometry revealed an expansion of the CD21-(low) B-cell population. This population was identified as the most reliable predictor of autoimmune cytopenias (15).

Specifically, the expansion of the CD21−CD24− naïve B-cell compartment that was observed in ITP patients may phenotypically resemble previously described Double-negative 2 (DN2) B cells (T−bet+CD21−) and Age-associated B Cells (ABCs) (34). These cells can be subdivided using classical B-cell markers, as well as others such as CD11c, FcRL4 and T-bet. These populations have been associated with chronic immune activation and extrafollicular B-cell responses in other contexts. It has been established that, bypassing the conventional germinal centre response, these cells are capable of expeditiously instigating autoimmunity, a mechanism frequently observed in SLE and rheumatoid arthritis (28, 29). Our findings suggest that ITP shares these extrafollicular signatures. However, our data are limited to phenotypic observations and do not demonstrate that these populations are functionally equivalent. Consequently, any relationship between CD21−CD24− B cells in ITP and DN2/ABC subsets, and their potential involvement in autoreactivity or defects in immune tolerance, must be considered hypothetical. Whether similar mechanisms operate in the ITP remains to be determined; since it remains unclear whether the expanded CD21–/low populations observed in different conditions (ageing and during chronic inflammatory conditions such as viral infections, malaria, common variable immunodeficiency, and autoimmune diseases) are truly equivalent, share the same gene expression profiles, or perform similar functions (34). However, recent single-cell atlas data from bone marrow in ITP patients (35) has identified the functional significance of the CD21− subset cells as a reservoir for autoreactivity, reflecting a profound defect in central B-cell tolerance. However, this interpretation is based on external data and was not directly assessed in our study.

In accordance with models of BAFF-driven tolerance breakdown (12) the markedly elevated plasma BAFF levels observed in the ITP cohorts, particularly among those aged >65—may function as a causative factor in pathogenesis of the disease. However, due to the cross-sectional design of the present study, it is not possible to infer causal relationships. It remains possible that increased BAFF levels are a secondary effect of chronic immune activation rather than a primary pathogenic mechanism. Despite the limitations of the present study, which is confined to peripheral blood phenotyping, the finding of expanded atypical subsets and elevated BAFF is highly consistent with developmental tolerance failures. This finding elucidates why the expansion of this specific subset serve as a reliable indicator of autoimmune pathology across different age groups. Nevertheless, it is important to note that it represents a snapshot of the systemic immune state and serve as indirect indicators of the complex processes occurring at primary tolerance checkpoints.

Flint et al. reported that a population of CD21− naïve B-cells expands in autoantibody-positive patients with ITP (36). Nevertheless, in our ITP cohorts, we did not observe these differences, possibly because our ITP cohorts are small or because the method used to detect antiplatelet antibodies is not sensitive enough to identify those with low affinity for platelet antigens.

Our results demonstrate differences in the distribution of B-cell subsets between ITP patients aged over 65 and those aged 65 or under, showing greater expansion of the CD21−CD24− naïve B-cell subset in older patients. While these results need to be validated in larger ITP patient cohorts, they have translational significance for determining the most appropriate therapy for ITP patients. New therapies for ITP are being developed that target B-cells. For B-cell depletion, rituximab and mycophenolate mofetil are recommended for the treatment of refractory ITP patients (6); or are being studied in clinical trials such as monoclonal antibodies directed against clusters of differentiation expressed by plasma cells such as CD38 (37); or are inhibitors of BAFF pathway or of Bruton kinase (5). Despite the efficacy of B-cell depletion therapies in ITP, frequent relapse implies de novo loss of tolerance early in B-cell development.

In conclusion, managing ITP in older patients is particularly important, as older age is associated with an increased risk of bleeding and a greater susceptibility to adverse effects of treatment. Our study deepens our understanding of the intersection between ageing and this autoimmune disease by identifying B-cell phenotypes that are specific to ITP and that could be targeted for treatment.

## Data Availability

The raw data supporting the conclusions of this article will be made available by the authors, without undue reservation.
