# Supplementary material for: Age-related and disease-specific changes in B-cell profiles in older adults with immune thrombocytopenia
Source: Front Immunol. 2026 May 12;17:1791460. doi: 10.3389/fimmu.2026.1791460 (PMC13201400; doi:10.3389/fimmu.2026.1791460)
Supplement: Supplementary file 1 [file DataSheet1.pdf]

Supplementary Material

1 Supplementary Table 1

Composition of the EuroFlow PIDOT and pre-germinal centre (GC) B cell tubes and information regarding the reference reagents included in it (van der Burg et al, Front Immunol. 2019 Mar 4;10:246. doi: 10.3389/fimmu.2019.00246; PMID: 30886612).

	**Fluorescent label**	**Antigens**	**Antibody, Cat. Number**
**Primary Immunodeficiency Orientation Tube (PIDOT)**	BV421	CD27	Becton Dickinson CYT-PIDOT8-27BV421
	BV510	CD45RA	Becton Dickinson CYT-PIDOT8-45RABV510
	FITC	CD8+/IgD	Becton Dickinson CYT-PIDOT8
	PE	CD16+/CD56	
	PerCP-Cyanine 5.5	CD4+/IgM	
	PE-Cyanine 7	CD19+/TCRγδ	
	APC	CD3	
	APC-C750™	CD45	
**B1 Pre-GC Tube**	BV421	CD27	Becton Dickinson CYT-PIDOT8-27BV421
	BV510	IgM	Biolegend 314521
	FITC	CD38	Becton Dickinson 340909
	PE	CD5	Biolegend 300608
	PerCP-Cyanine 5.5	IgD	Biolegend 348208
	PE-Cyanine 7	CD19	Beckman Coulter IM3628
	APC	CD21	Becton Dickinson 559867

	APC-Alexa Fluor 750	CD24	Beckman Coulter B10738

Del Pino-Molina L, López-Granados E, Lecrevisse Q, Torres Canizales J, Pérez-Andrés M, Blanco E, Wentink M, Bonroy C, Nechvatalova J, Milota T, Kienzler AK, Philippé J, Sousa AE, van der Burg M, Kalina T, van Dongen JJM, Orfao A. Dissection of the Pre-Germinal Center B-Cell Maturation Pathway in Common Variable Immunodeficiency Based on Standardized Flow Cytometric EuroFlow Tools.Front Immunol. 2021 Feb 17;11:603972. doi: 10.3389/fimmu.2020.603972.PMID: 33679693

Supplementary Figure 1- To study B cell distribution, we used a tube designed by the EuroFlow group, following their guidelines for gating strategy, compensation and analysis. In a first step, total B cells were gated as FSClo/SSClo/CD19+ (A). In a second step (B), B cell were dissected into 9 subpopulations based on their staining profile for CD19, CD38, CD24, CD21, CD27, CD5, surface membrane IgM and IgD. Unsw: Unswitched. Sw: Switched. MBC: Memory B cells. Pb: Plasmablasts.

As a demonstrative example, we present here a case of the gating of the LB population of a patient with ITP>65.

B Cell identification: B-cells were identified according to their forward and side scatter properties and positive CD19 expression (highlighted in the pink region)

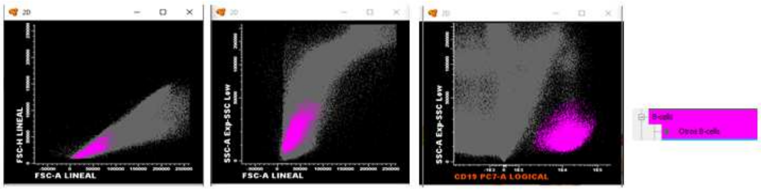

Plasma cell identification: Plasma cells were identified based on their light-scatter properties, which are low to intermediate and position them between lymphocytes and monocytes. They were also identified by their immunophenotype, which is characterised by dim CD19 and bright CD38 expression in the absence of CD5, CD21 and CD24 (red gate).

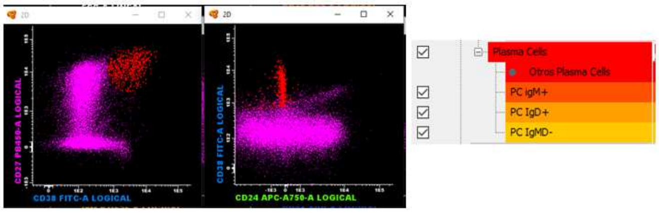

Subsequently, plasma cells were subclassified into IgM$^+$, IgD$^+$, and other plasma cell subsets.

Non-switched memory B-cells: Non-switched memory B cells were recognized by their low light-scatter characteristics typical of lymphocytes, with co-expression of CD19 and CD27, and surface IgM/IgD positivity, in the absence of CD5 and CD38 (green gate).

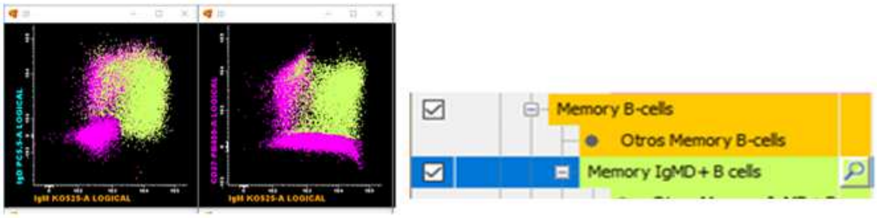

Switched (smIgMD-) Memory B-cells gating strategy: Switched memory B cells were identified based on their low light-scatter characteristics, typical of lymphocytes, and expression of CD19, in the absence of CD5, CD38, and surface IgM/IgD (smIgM/smIgD) (blue gate).

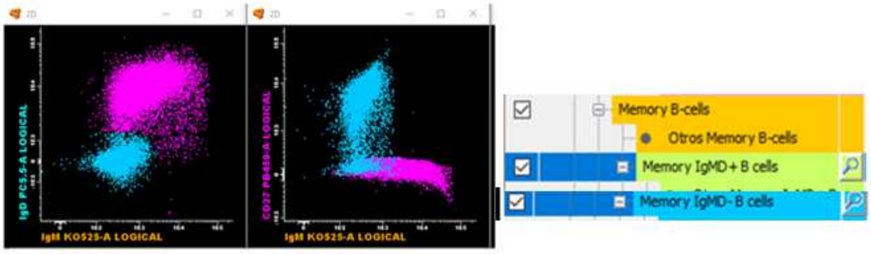

Pre-germinal-centre B cell gating strategy: Pre-germinal-centre (GC) B cells were identified by their low light-scatter properties (in the lymphocyte region) and co-expression of CD19 with surface IgM and IgD, in the absence of CD27.

Following the normal pathway of B cell maturation, the distinction between immature/transitional and naïve B-cell subsets was based on the expression profile of CD5 and CD38.

These naïve B-cell subsets were further characterized using CD21 and CD24 expression patterns.

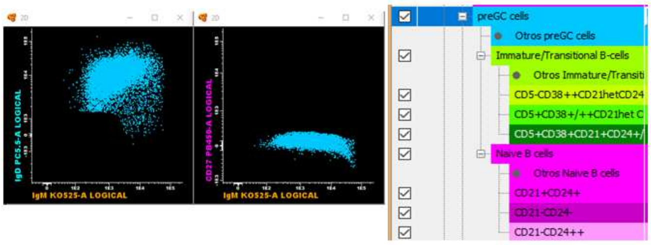

Immature/Transitional B cells: Immature or transitional B cells were defined as those with high CD38 and CD24 expression, including subsets that are CD5^{+} (green gate).

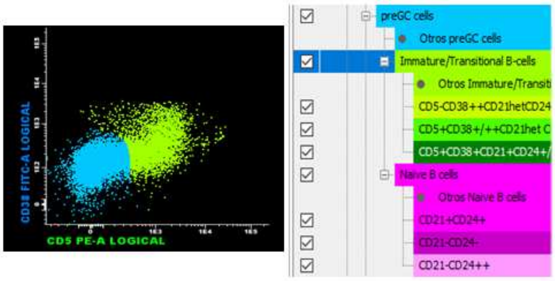

According to their maturation stage, three phenotypic subsets were identified:

1. CD5^{-}, CD38^{++}, CD21het, CD24^{++}
2. CD5^{+}, CD38^{+}/$^{++}$, CD21het, CD24^{++}
3. CD5^{+}, CD38^{+}/$^{++}$, CD21^{+}, CD24

1. CD5 –CD38++CD21hetCD24++:

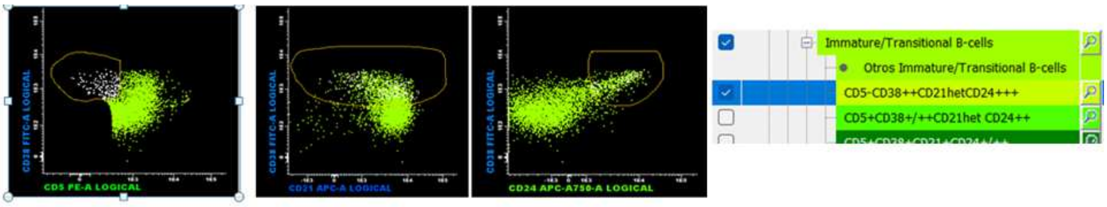

2. CD5+CD38+CD21hetCD24++:

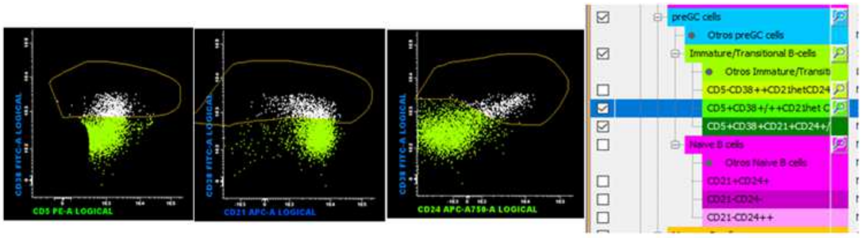

3. CD5+CD38+CD21hetCD24+:

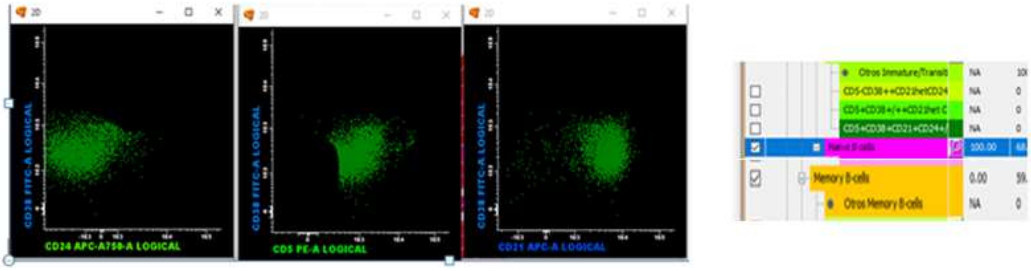

Naïve B cells: These were defined as mature pre-germinal-centre (pre-GC) B cells that lacked the expression of CD38 and CD5.

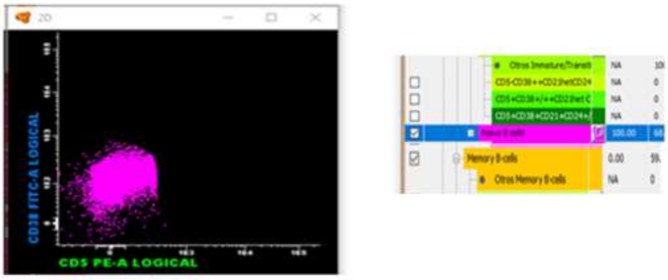

Within this compartment, three subsets of mature naïve B cells were distinguished according to their CD21 and CD24 expression profiles as follow:

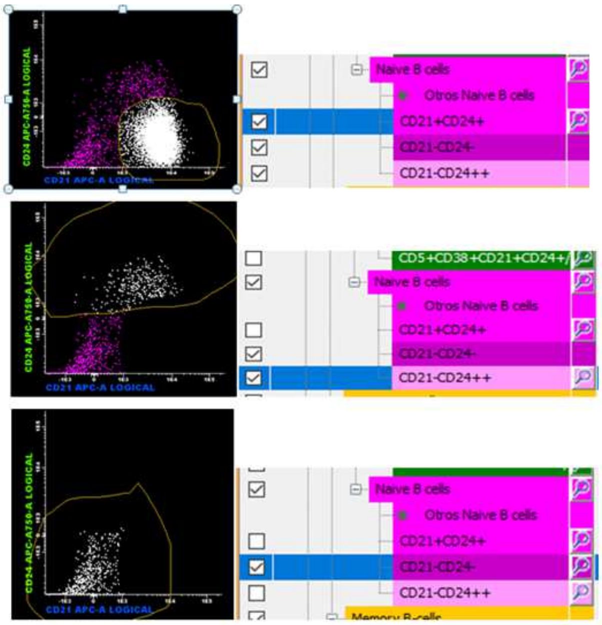

As the greatest difference between ITP<65 and ITP>65 in the distribution of LB subsets is found in the CD21-CD24- group of naïve B cells, we present below the dot plots of the total naïve cell population and the naïve CD21-CD24- population of one HC>65, one ITP>65 and one ITP≤65. The proportion of CD21-CD24- cells in the total naïve B cell population was: HC>65: 1.02%; ITP>65: 11.2%; ITP≤65: 2.5%.

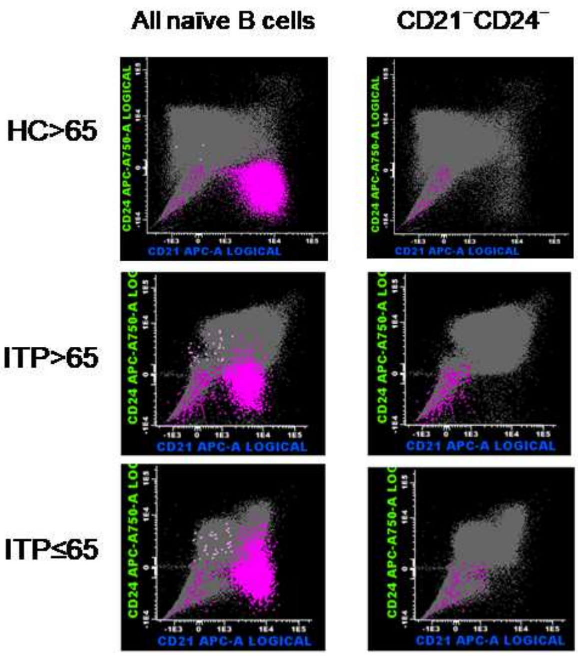

The gating strategy was previously published in the Supplementary Figure 1 from del Pino Molina L et al, "Dissection of the Pre-Germinal Center B-Cell Maturation Pathway in Common Variable Immunodeficiency Based on Standardized Flow Cytometric EuroFlow Tools", DOI: 10.3389/fimmu.2020.603972."

3- Supplementary Table 2

	≤65 yo [Median (p25-p75)]	>65 yo [Median (p25-p75)]	P value
B-cells	117 (60-228)	86 (53-177)	0.3617
CD5-CD38++CD21hetCD24+++	1.22 (0.52-3.35)	0.56 (0.31-1.18)	0.0294*
CD5+CD38+/++CD21het CD24++	3.78 (1.91-9.07)	1.12 (0.38-4.34)	0.0155*
CD5+CD38+CD21+CD24+/++	8.58 (5.65-16.70)	4.33 (1.86-11.76)	0.0125*
CD21+CD24+	41.75 (26.22-83.60)	20.32 (13.58-85.78)	0.2594
CD21-CD24-	1.16 (0.97-2.05)	2.22 (1.34-3.50)	0.0257*
CD21-CD24++	0.16 (0.09-0.23)	0.25 (0.11-0.44)	0.0896

Memory IgMD+ B cells	15.42 (6.56-23.13)	17.92 (8.54-33.17)	0.4891
Memory IgMD- B cells	20.09 (6.47-31.98)	16.25 (11.33-30.66)	0.8769
Plasma Cells	1.64 (0.68-4.57)	1.085 (0.40-2.05)	0.1048
BAFF (pg/mL)	601 (453-778)	804 (598-1640)	0.046*

Distribution of distinct subsets of pre-germinal centre (GC) and post-GC B-cells determined by flow cytometry and plasma BAFF levels by ELISA. A comparison was made between ITP≤65 yo and ITP>65 yo patients treated in monotherapy with TPO-RAs.A Mann–Whitney test was performed on the ITP≤65 yo and ITP>65 yo groups, and a p-value<0.05 was considered significant. Denotes significance.*
